# Long Island Veterinary Association

**Published:** 1891-02

**Authors:** D. S. Breslin

**Affiliations:** Secretary


*Long Island Veterinary Society.*—A regular meeting of the Long Island Veterinary Society was held on January 21, 1891, at No. 74 Adam St., the President, Dr. Roscoe R. Bell in the chair.

The following members were present, Drs. Geo. H. Berns, Roscoe R. Bell, Geo. F. Bowers, H. Housman, Samuel Atchison, D. S. Breslin, Philip Newman, Wm. H. Pendry. The minutes of the previous meeting were read and approved.

The Board of Censors made no report.

The next order of business, reading of papers, the secretary read a communication from the essayist Dr. Geo. G. Vanderveer, stating his inability to attend the meeting but forwarding paper on Leukaemia. It was moved and seconded that the communication be received and that the secretary read the paper. Carried

After the reading of the paper it was moved and seconded that the discussion of the paper be postponed until the next meeting. Carried.

The meeting then adjourned.

D. S. BRESLIN, *Secretary.*

